# The comparative risk of acute kidney injury of vancomycin relative to other common antibiotics

**DOI:** 10.1038/s41598-020-73687-9

**Published:** 2020-10-14

**Authors:** Martina Gaggl, Virginia Pate, Til Stürmer, Abhijit V. Kshirsagar, J. Bradley Layton

**Affiliations:** 1 Department of Epidemiology, Gillings School of Global Public Health, University of North Carolina, 135 Dauer Drive, 2101 McGavran-Greenberg Hall, CB #7435, Chapel Hill, NC USA; 2 Division of Nephrology and Dialysis, Department of Medicine III, Medical University of Vienna, Währinger Gürtel 18-20, Vienna, Austria; 3 Division of Nephrology and Hypertension, UNC Kidney Center, University of North Carolina, Chapel Hill, NC USA; 4 RTI Health Solutions, Research Triangle Park, NC USA

**Keywords:** Microbiology, Health care, Nephrology

## Abstract

The glycopeptide antibiotic vancomycin is a mainstay in the treatment of Gram-positive infection. While its association with acute kidney injury (AKI) has waxed and waned, recent data suggest nephrotoxicity, even as mono-therapy. Our study aimed to evaluate the 2-week risk of AKI after at least 3 days of intravenous vancomycin mono-therapy initiated within 5 days of hospitalization compared to other intravenous antibiotics used for similar indications. We used a new user-active comparator study design and identified patients with a first hospitalization during which they received vancomycin or comparator, from commercial claims based in the United States. We estimated incidence rates, hazard ratios using adjusted cox-regression models, and standardized mortality/morbidity ratio weighted cox-regression models. In the 32,997 patients vancomycin was used in 17% of patients and 129 cases of AKI were observed. Overall incidence of AKI was 9.3 (95% CI 0.78–1.22) per 100 person-years. The adjusted hazard ratio for vancomycin versus all other comparators was 0.74 (95% CI 0.45–1.21). Separate models for respective comparators resulted in hazard ratios below the null, except for vancomycin vs. cefazolin. Intravenous vancomycin mono-therapy does not increase the risk of AKI compared to other intravenous antibiotics used for similar indication in this cohort of hospitalized patients.

## Introduction

Vancomycin has been the major antibiotic treatment for Gram-positive infections since the emergence of methicillin resistant *Staphylococcus aureus* in the 1970s. From 2006 through 2012, it was among the most frequently used antibiotics in the United States (US)^1^. While a high frequency of acute kidney injury (AKI) was reported with early formulations of vancomycin, it was felt to be due primarily to impurities^2^. At conventional doses, Vancomycin is believed to be kidney safe, and any observed kidney injury may be due to a combination of patient characteristics and comorbidities, vancomycin dose, concomitant medication use, and varying definitions of AKI^3^.

A recent meta-analysis from Ray et al*.* has raised concerns about kidney injury from vancomycin monotherapy^4^. Including cohort studies and randomized controlled trials, it demonstrated a greater than a two-fold risk of developing AKI for vancomycin monotherapy compared to other single agents. The majority of the studies included linezolid, known to have no kidney toxicity, as the comparator^4^. The authors concluded that in vancomycin treated patients about one half of AKI cases could be attributed to vancomycin treatment (attributable risk 59%).

Given the widespread use of vancomycin and the potential impact of the recent findings, we sought to corroborate the results in a contemporary cohort of adults assembled from a large commercial claims database in the US. Our study evaluated the 2-week risk of AKI in patients after at least 3 days of intravenous (IV) vancomycin mono-treatment compared to patients treated with a comparator for similar clinical indications.

## Material and methods

We used the MarketScan Research Databases (IBM Watson Health), which consist of the Commercial Claims and Encounters data set–employer-based commercial insurance plans for employees, spouses and dependents aged younger than 65 years from large US insurers—and the Medicare Supplementary and Coordination of Benefit data set—employer-based Medicare supplementary insurance for patients aged 65 or older. The Institutional Review Board of the University of North Carolina at Chapel Hill approved the study and all methods were carried out in accordance with relevant guidelines and regulations.

**Figure 1:**
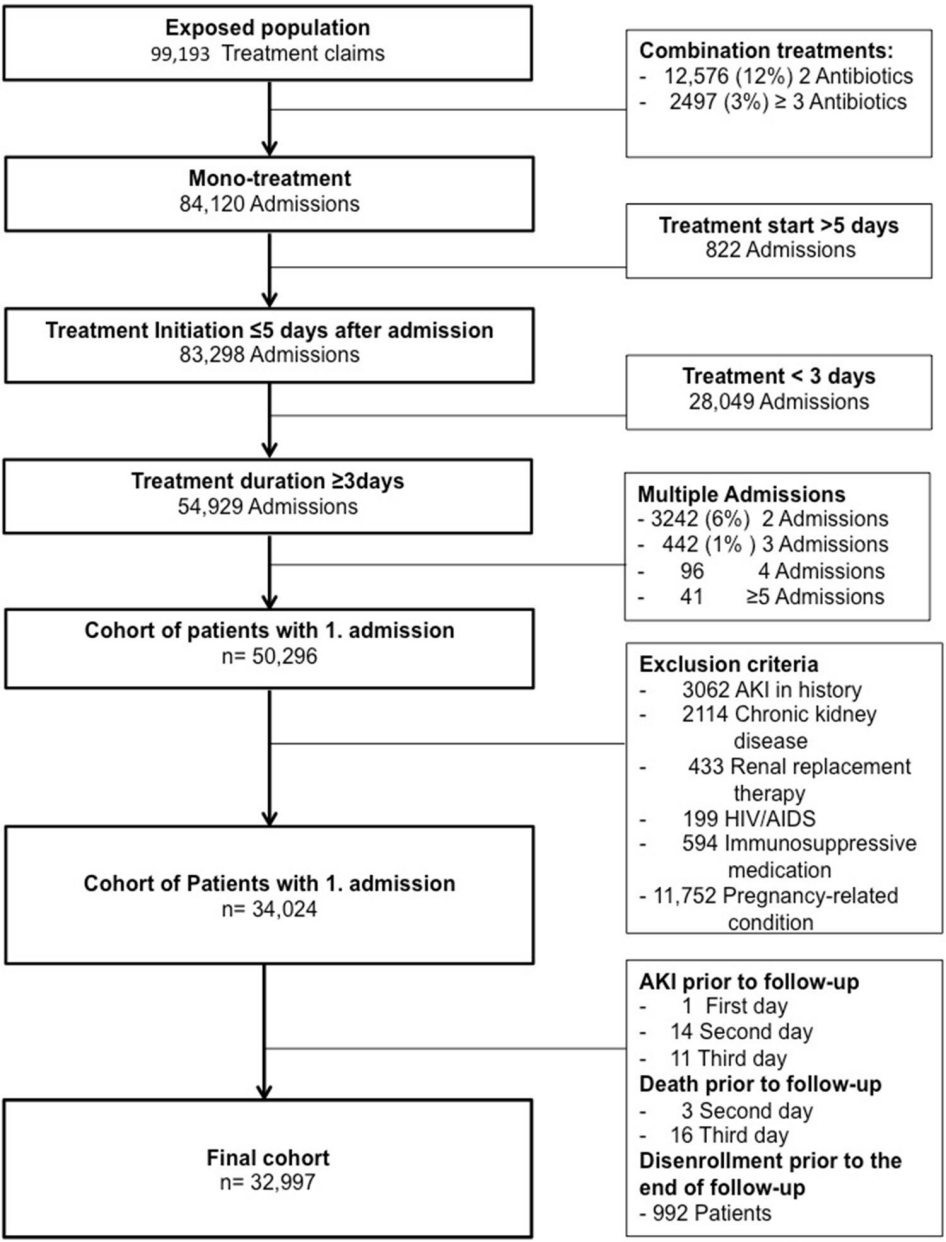
Study design.

We identified the first hospitalization per patient treated with IV mono-therapy with either vancomycin or comparator treatment for at least 3 days with a treatment onset within 5 days of hospitalization between January 2000 and October 2015. The cohort was restricted to patients with at least 1 year of previous continuous enrolment (Fig. 1). In a sensitivity analysis we restricted the cohort to subjects with a treatment for at least 5 days and performed identical analysis as detailed below.

Subjects with the following conditions within 12 months before the index treatment were excluded: history of AKI; chronic kidney disease (including end-stage renal disease, dialysis, and renal transplant) since renal impairment increases AKI risk; human-immunodeficiency virus/acquired immunodeficiency syndrome or use of immunosuppressive medication (cyclosporine, mycophenolate mofetil, tacrolimus, azathioprine, sirolimus, and everolimus) since chronically immunosuppressed patients develop opportunistic infections requiring special treatments regimes that are incomparable with non-immunosuppressed patients. Furthermore, we excluded women with pregnancy-related conditions or procedures since they almost exclusively received cefazolin, and pregnancy-related AKI is most likely due to other reasons.

### Exposure

To identify treatment we used health care common procedure codes (HCPC) for vancomycin treatment and comparator substances (linezolid, cefazolin, cefepime, piperacillin + tazobactam, meropenem, ertapenem, daptomycin) (supplemental Table 1a).

HCPC codes occurring on consecutive days were considered one treatment period; gaps in treatment were permitted, and the treatment period lasted from the first claim occurring until the last claim occurring during the hospitalization. Subsequently each patient had one treatment period per type of treatment and admission.

Patients with combination treatments were excluded from the analysis. Combination treatment was defined as concurrently starting more than one antibiotic treatment of interest at the same day. Patients with any further antibiotic treatment during the same hospitalization starting after the first day of treatment exposure were not excluded.

Duration of treatment was determined from the procedure claim and categorized in “ < 7 days of treatment”, “7 to 14 days of treatment”, and “ ≥ 15 days of treatment” for further analysis.

### Outcome

The outcome was AKI within 14 days after ≥ 3 day period of IV vancomycin treatment or comparator treatment. Day 4 of treatment was considered the index date when the 14-day follow-up period started (Fig. 1). Our AKI outcome definition included inpatient or outpatient diagnoses of acute renal failure or kidney failure, or a procedure code for dialysis in any position^5^ (supplemental Table 1b).

### Covariates

We assessed comorbidities and co-medications in the claims during the 12 months before the treatment initiation using ICD-9-CM diagnosis and procedure codes and 11-digits national drug codes (NDC), including: other kidney disease, proteinuria, kidney stones, hypercalciuria, diabetes mellitus, cardiovascular disease, hypertension, hyperlipidemia, atrial fibrillation, heart failure, chronic liver disease and cirrhosis, multiple myeloma, systemic lupus erythematous (supplemental Table 1c). Co-medications included angiotensin-converting enzyme inhibitors (ACEi), angiotensin 2 receptor antagonists (ARB), statins, beta-blockers, calcium channel blockers, antiplatelet agents, alpha-blockers, thiazide, K^+^-sparing diuretics, loop diuretics, niacin, fibrates, ezetimibe, anticoagulants, non-steroidal anti-inflammatory drugs (NSAID), and steroids.

Using inpatient diagnosis information, hospitalizations were categorized as either primarily due to infection or some other reason. First, we classified diagnoses into broad diagnosis groups as suggested by CCS Healthcare Cost Utilization Project (HCUP) group codes (table S1d)^6^. Second, if an infection was recorded (“sepsis”, “skin infection”, or “other infection”) in any diagnosis position, this code was considered the main diagnosis. Otherwise the inpatient primary diagnosis field information was used.

To control for surgical procedures, procedure codes were grouped as suggested by Grams et al*.* as major (ear-nose-throat, thoracic, cardiac, vascular, general, urology, orthopedic)^7^, minor (procedure labels including terms in supplemental table S1, if not classified as major surgery)), or none.

### Follow-up time and censoring

Patients were followed for 14 days beginning form the index date (fourth day of treatment) and administratively censored at the end of day 14, if they did not have the outcome event. Furthermore, patients were censored if they died in the hospital. Because death information was limited to in-hospital deaths, we assumed complete follow-up after discharge for each patient unless there was insurance plan disenrollment.

### Statistical analysis

We estimated descriptive statistics, including means and standard deviations (SD), and frequencies. Incidence rates and their 95% confidence intervals (95% CI) were estimated for the overall cohort and individual treatment groups^8^.

We estimated hazard ratios (HR) and 95% CIs with cox-regression models comparing the vancomycin group against all other comparators combined (reference group), and separately for each comparator (reference group). To account for covariate imbalances between treatment groups we, first, constructed cox-regression models adjusted for age, gender, and main diagnosis categories (“cardiac” and “thoracic” combined as “cardiothoracic”; “urology”, “vascular”, and “ENT” combined as “other surgeries”). For models comparing ertapenem, daptomycin, and cefepime against vancomycin, respectively, the main diagnosis categories were replaced by a binary infection indicator, because of low cell counts. Second, we estimated propensity scores (PS), to account for confounders that might have influenced the choice of treatment. To directly compare the estimates across the vancomycin and comparator group we standardized the covariate distribution of the comparators to the covariate distribution of vancomycin treated patients using standardized mortality/morbidity ratio (SMR) weights [PS/(1-PS)]^9^. SMR weighting creates comparator cohorts with the same covariate distributions as in the vancomycin group. We calculated the SMR-weighted (adjusted) hazard ratio and 95% CIs for vancomycin *vs.* comparators using Cox regression.

Furthermore, we stratified the follow-up as less than 7 days, 7–14 days, and more than 14 days of antibiotic treatment.

The assumption of proportional hazards was examined visually, and ties were controlled using the Efron method. All statistical analyses were preformed with SAS 9.2 (SAS Institute, Inc, Cary, NC).

## Results

**Figure 2:**
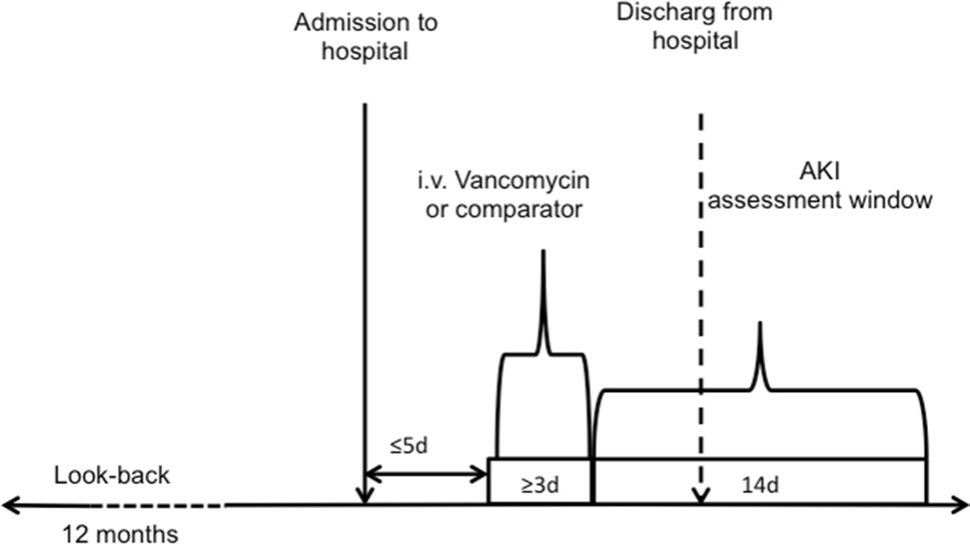
Study Cohort.

Between 2000 and October 2015, we identified 32,997 patients with a hospitalization with vancomycin or comparator mono-therapy, meeting the inclusion criteria (Fig. 2). The mean age was 50 (SD 15) years and 19,800 (60%) patients were female. Vancomycin was used in 5449 (17%) patients, one of the comparators was administered in the remaining 27,548. The mean treatment duration was 5.3 (± 4.7) days, and treatment started on the first day of hospitalization in 99% of patients. Comorbidities and co-medications assessed in the 1-year-look-back period are presented in Table 1. Overall, 15% of the hospitalizations included a specific infection diagnosis code, and surgical procedures were performed in 15,760 (48%) of patients (supplemental Table 2).

**Table 1:** Patient’s characteristics.

	Vancomycin		Comparator	
N	5449	16.5%	27,548	83.5%
Gender (female)	2945	14.9%	16,855	85.1%
Age (years)	50.4	± 15.7	50.2	± 14.9
**Treatment duration**				
≤ 7 days	4347	79.8%	23,965	87.0%
8–14 days	871	16.0%	2845	10.3%
≥ 15 days	231	4.2%	738	2.7%
Infection coded (yes)	2242	41.1%	2785	10.1%
Surgical procedure (yes)	1965	36.1%	13,795	50.1%
Cardiovascular	246	4.5%	696	2.5%
Heart disease	710	13.0%	2210	8.0%
Diabetes	1646	30.2%	6567	23.8%
Hyperlipidemia	2231	40.9%	10,473	38.0%
Hypertension	2905	53.3%	13,251	48.1%
Cirrhosis	256	4.7%	1310	4.8%
Atrial fibrillation	344	6.3%	1241	4.5%
Hypercalemia	79	1.5%	446	1.6%
Lupus	155	2.8%	566	2.0%
Myeloma	26	0.5%	70	0.3%
Nephrolithiasis	186	3.4%	1051	3.8%
ACEi	1003	18.4%	4526	16.4%
ARB	544	10.0%	2660	9.7%
Statin	1200	22.0%	5499	20.0%
Beta-blocker	988	18.2%	4304	15.6%
Ca-channel-blocker	651	12.0%	3004	10.9%
Alpha-blocker	215	3.9%	894	3.2%
Thiazide	707	13.0%	3881	14.1%
K^+^-sparing diuretics	244	4.5%	1197	4.3%
Loop diuretic	438	8.0%	1387	5.0%
Niacin	63	1.2%	214	0.8%
Fibrate	172	3.2%	809	2.9%
Ezetimibe	131	2.4%	610	2.2%
OAK	604	11.1%	2315	8.4%
Aspirin	51	0.9%	200	0.7%
NSAID	1511	27.7%	7671	27.8%

*ACEi* angiotensin converting enzyme inhibitor, *ARB* angiotensin receptor2 blocker, *Ca* channel-blocker, calcium channel blocker, *OAK* oral anticoagulation, *NSAID* non-steroidal anti-inflammatory drug.

Within the 14-day follow-up period, we observed 129 cases of AKI. Seventy-two (56%) were male, 60 (47%) had a surgical procedure, and the most frequent indications were sepsis (15%) and gastrointestinal disease (14%). The total follow-up was 493,362 person-days, and 99.4% of patients completed all 14 days of follow-up.

In-hospital death occurred in 93 patients (0.2%; 6% missing discharge status).

Overall, the incidence of AKI was 9.3 cases (95% CI 7.8–12.2) per 100 person-years, 9.6 (95% CI 6.3–14.6) per 100 person-years in the vancomycin group and 9.3 (95% CI 7.7–11.2) per 100 person-years for the comparator group. Detailed incidence rates in each comparator substance are given in Table 2.

**Table 2:** Incidence rates and hazard ratios of vancomycin compared to all comparators and vancomycin compared to singular comparator.

	N	Cases	Person time^b^	Incidence rate^b^	95% CI	Crude HR	95% CI	Adjusted HR^c^	95% CI
Vancomycin^a^	5449	22	22.35	0.93	0.94–1.92				
Comparators	27,548	107	113.93	0.96	0.77–1.12	1.04	0.85–1.90	0.74	0.45–1.21
Cefazolin	20,124	58	83.31	0.69	0.53–0.89	1.40	0.86–2.29	1.01	0.55–1.83
Linezolid	251	6	1.03	5.76	2.59–12.83	0.17	0.07–0.42	0.17	0.01–0.46
Piperacillin	3885	25	16.00	1.54	1.04–2.28	0.63	0.35–1.11	0.47	0.24–0.91
Cefepime	433	2	1.79	1.11	0.28–4.43	0.87	0.45–3.70	0.80	0.19–3.69
Meropenem	379	7	1.58	4.44	2.12–9.32	0.22	0.09–0.51	0.21	0.10–0.52
Ertapenem	2205	8	9.13	0.86	0.43–1.73	1.11	0.51–2.50	0.47	0.19–1.17
Daptomycin	271	1	1.12	0.88	0.12–6.25	1.09	0.15–8.11	0.99	0.12–8.19

*95% CI* 95% confidence interval, *HR* hazard ratio.

^a^Index group.

^b^Per 10 person-years.

^c^Adjusted for age, gender, medical history, co-medication, type of surgery, main diagnosis (not Included in model because of low cell count: vancomycin vs. piperacillin ,vancomycin vs. ertapenem, vancomycin vs. daptomycin, and vancomycin vs. cefepime).

After multivariable adjustment, the hazard ratio for vancomycin *vs.* all other comparators was 0.74 (95% CI 0.45–1.21) (Table 2). Similarly, separate adjusted models for respective comparators resulted in individual hazard ratios below the null, except for vancomycin vs. cefazolin (HR = 1.01, 95% CI 0.55–1.83). Notably, the association between vancomycin and AKI when compared to linezolid or meropenem remained basically unchanged after adjustment for confounding. The hazard ratio for vancomycin vs. piperacillin became stronger (HR = 0.47, 95% CI 0.24–0.91; Table 2) in the adjusted model.

The SMR-weighted cox-model resulted in a comparable hazard ratio of 0.70 (95% CI 0.41–1.20) for vancomycin *vs.* all comparators.

Since a longer time of treatment with vancomycin is described as an important risk factor for AKI, we stratified the overall model for treatment duration (Table 3). In the adjusted model vancomycin treatment for more than 6 days was associated with a lower hazard to develop AKI than comparators (7–14 days: HR = 0.89, 95% CI 0.37–2.12; > 14 days: HR = 0.89, 95% CI 0.26–3.10), but the effect is attenuated compared to the overall adjusted hazard ratio (HR = 0.74, 95% CI 0.45–1.21). Notably, all confidence intervals are crossing the null in stratified models.

**Table 3:** Incidence rates and hazard ratios of vancomycin compared to all comparators stratified for antibiotic treatment duration.

	N	Cases	Person time^b^	Incidence rate^b^	95% CI	Crude HR	95% CI	Adjusted HR^c^	95% CI
**< 7 days of treatment**									
Vancomycin^a^	4347	8	18.25	0.44	0.22–0.88				
Comparators	23,965	72	100.67	0.72	0.57–0.90	0.61	0.30–1.27	0.49	0.22–1.08
**7 to 14 days of treatment**									
Vancomycin^a^	871	9	3.64	2.47	1.28–4.75				
Comparators	2845	23	11.88	1,94	1.29–2.91	1.28	0.59–2.76	0.89	0.37–2.12
**> 14 days of treatment**									
Vancomycin^a^	231	5	0.96	5.19	2.16–12.48				
Comparators	738	12	3.08	3.89	2.21–6.86	1.34	0.47–3.79	0.89	0.26–3.10

*95% CI* 95% confidence interval, *HR* hazard ratio.

^a^Index group.

^b^Per 10 person-years.

^c^Adjusted for age, gender, medical history, co-medication, type of surgery, main diagnosis;

As a sensitivity analysis, we restricted to patients with treatment with the index antibiotic for at least 5 days and follow-up was from day 6 until 14. In general, we observed similar results (Table 4) and the conclusions remained unchanged.

**Table 4:** Incidence rates and hazard ratios of vancomycin compared to all comparators and vancomycin compared to singular comparator, restricted to a cohort with at least 5 days of antibiotic treatment.

	N	Cases	Person time^b^	Incidence rate^b^	95% CI	Crude HR	95% CI	Adjusted HR^c^	95% CI
Vancomycin^a^	2959	14	12.41	1.13	0.67–1.91				
Comparators	10,652	63	44.61	1.41	0.10–1.81	0.80	0.45–1.43	0.62	0.34–1.15
Cefazolin	6426	26	26.97	0.96	0.66–1.42	1.17	0.61–2.24	0.84	0.38–1.83
Linezolid	210	6	0.87	6.91	3.11–15.39	0.16	0.06–0.43	0.13	0.05–0.40
Piperacillin	2097	20	8.74	2.29	1.48–3.55	0.49	0.25–0.98	0.35	0.15–0.82
Cefepime	276	1	1.15	0.87	0.12–6.18	1.30	0.17–9.86	1.23	0.13–11.39
Meropenem	278	4	1.16	3.44	2.29–9.16	0.33	0.11–1.00	0.34	0.10–1.20
Ertapenem	1174	5	4.92	1.02	0.42–2.44	1.11	0.40–3.1	0.40	0.12–1.27
Daptomycin	191	1	0.80	1.25	0.18–8.90	0.90	0.12–6.85	0.68	0.08–5.81

*95% CI* 95% confidence interval, *HR* hazard ratio.

^a^Index group.

^b^Per 10 person-years.

^c^Adjusted for age, gender, medical history, co-medication, type of surgery, main diagnosis (not Included in model because of low cell count: vancomycin vs. piperacillin ,vancomycin vs. ertapenem, vancomycin vs. daptomycin, and vancomycin vs. cefepime).

## Discussion

In this large contemporary cohort of 32,997 hospitalized patients with various clinical indications for IV antibiotic treatment, we compared the risk of AKI associated with IV vancomycin mono-therapy to other IV antibiotics commonly used for Gram-positive infections. After adjustment for demographic and medical characteristics, the analysis did not demonstrate an association between IV vancomycin and AKI relative to the other IV antibiotics. We observed trends towards a decreased number of AKI cases attributed to treatment with vancomycin compared individually to linezolid, piperacillin/tacobactam, meropenem, ertapenem, and daptomycin; however, these estimates were all based on small numbers of outcomes. Compared with cefazolin treatment, the most commonly identified IV antibiotic, we observed a minimally elevated hazard for AKI in the vancomycin group that did not reach statistical significance.

The common belief that vancomycin is nephrotoxic derives from early experiences with impure formulations of the drug^3^, observational studies with methodological limitations^10,11^, and cohort studies that were originally not designed to assess renal outcomes^12–15^. In animal models, vancomycin causes oxidative stress in the proximal tubule^16^ and possibly also in the distal tubule^17^, which would suggest dose-dependent acute toxic tubular injury in the human setting. In contrast, most published case reports describe acute interstitial nephritis after vancomycin treatment, suggesting an allergenic, dose-independent mechanism of AKI (reviewed in Ray et al.^4^). Although this contradiction might be due to publication bias, it introduces further controversy in the question of whether vancomycin is nephrotoxic.

Most observational studies analyzed small cohorts and were limited to vancomycin users without a control group^10,11^. Our study included new-users of vancomycin compared to an active comparator group of other, similar IV antibiotics. Restricting to the first treatment period mitigates bias of over-selection of patients “immune” to the outcome, while the active comparators with comparable clinical indications for the treatment controls for indication bias^18^. We chose comparators with similar bactericidal activity (linezolid, cefazolin, daptomycin) or clinical indications (piperacillin/tacobactam, cefepime, meropenem, ertapenem) to build a comparator group with an approximate equal baseline risk of AKI compared to the vancomycin group^19^.

Cohort studies, most of which used linezolid as control, were not matched for baseline risk of AKI and thus might have drawn misleading conclusions. Linezolid use in the commercial claims data is limited, but the small subsample of patients treated with linezolid had the highest incidence rate of AKI, although high-risk patients with CKD were already excluded from our analysis.

In contrast to the here-described study, several prior studies limited their study population to methicillin resistant *Staphylococcus aureus* (MRSA) infections or specific locations of infection, such as skin infection or pneumonia. The randomized controlled trial by Wunderink et al*.* randomized 1225 subjects and primarily assessed microbiologic response after the end of treatment between linezolid and vancomycin treated patients^12^. They showed that the renal toxicity was greater in the vancomycin group when day 3-trough levels were above 35 µg/mL, which was true for subjects with reduced baseline renal function and normal renal function. All subjects simultaneously received a Gram-negative antibiotic and patients with a mixed infection with the Gram-negative bacteria being the dominant pathogen were discontinued, as well as patients with Gram-negative pathogens resistant to the empirical treatment.

While restricting on treatment indications produced comparable cohorts across randomized trials, exclusion criteria varied substantially and produced rather unique cohorts. Most of the studies lacked information on concomitant antibiotic medication, which however might have also contributed to AKI.

More recent research on nephrotoxicity and vancomycin focused on antibactericidal combination treatments^20^. Especially the combination of piperacillin/tazobactam with vancomycin increased the risk of AKI compared to both, vancomycin and piperacillin mono-treatments^21^, but also compared to combination treatment of cefepime and vancomycin^22^, or meropenem and vancomycin^23^. This knowledge raises additional concerns about inferences made from previous studies evaluating vancomycin mono-treatment, as none of them accounted for or excluded concomitant use of other antibiotics.

Several risk factors for vancomycin use and AKI have been proposed. Prolonged duration of treatment with vancomycin is associated with an increased risk of AKI^11,24,25^. In our study, treatment duration was similar across all antibiotic groups, and the treatment duration was independent of received treatment associated with AKI, suggesting that duration of the antibiotic treatment is more a mediator for severity of infection and overall poor clinical status with higher AKI baseline risk.

Another such risk factor associated with AKI is high vancomycin trough levels^10,12,26–29^. Close therapeutic drug monitoring is recommended for vancomycin to increase clinical efficacy and avoid nephrotoxicity and commercial assays to measure through levels are widely available^30,31^. Alternatively, the high trough may be a result of an acute loss of kidney function^3^, as 90% of vancomycin is renally excreted^32^. Although, information on vancomycin through levels would have informed our analysis, it is very likely that under standard treatment protocols, toxic overdosing with subsequent adverse outcomes such as nephrotoxicity was also present in the comparator group. Therapeutic drug monitoring would also increase efficacy and minimize harm in other antibiotic classes^33^. Recent studies showed that under-dosing and over-dosing in selected patient groups, such as critically-ill, elderly, burn patients and severely obese patients, occurs also with standard dosing of beta-lactam antibiotics^34^. Pea et al*.* showed that over-exposure occurred in 33% of patients with standard doses of linezolid, and they suggest that drug monitoring would be a valuable approach to avoid toxicity^35^. Given that evidence, including information on vancomycin through levels in our analysis would have created missing information on through levels of comparators. The policy of continuous vancomycin infusion vs. intermittent application has been proposed to increase efficacy and safety^36,37^; since HCPCS codes are used for individual doses, our analysis cannot account for this treatment strategy. However, this strategy has also been discussed for other antibiotic treatments^38,39^.

Our analysis stands in contrast to previous studies, particularly for users of cefazolin, which is commonly used for minor infections with Gram-positive bacteria or as prophylactic treatment; however, cefazolin did have approximately the same number of AKI cases compared to vancomycin users, which is usually used for more severe infections.

When we restricted to a cohort with at least 5 days of antibiotic treatment, likely excluding the majority of prophylactic treatments, the adjusted model suggested that vancomycin users had a lower hazard of AKI than cefazolin users. The label of the current cefazolin formulation states nephrotoxicity and renal failure (unknown frequency) as a potential adverse event^40^, identical to the current drug information of vancomycin^41^. Regardless of the stated drug information of both substances, the nephrotoxic potential of vancomycin is perceived as much more significant in clinical practice, and this is probably true for all comparator classes that we assessed in this study.

This study has several limitations: First, as with all administrative claims-based studies, measures of renal function like AKI or CKD are insensitive^42,43^, suggesting the potential for outcome misclassification. Furthermore, previous studies of community-acquired AKI have demonstrated a comparable, low frequency of AKI despite the use of serum creatinine to estimate function^44–47^. However, measures of relative effect, such as the reported hazard ratios, should still be unbiased^48,49^ given the high specificity of billing codes for AKI^42^; thus we report relative, rather than absolute measures of effect. However, residual bias is still possible.

Second, restricting to a 3-day exposure window is arbitrary and could introduce selection bias, but is commonly applied in prior studies. We argue that patients developing AKI after one or two doses of vancomycin, might have underlying conditions rendering AKI more likely. By restricting to a slightly longer period of use, the possibility of a causal effect observed due to vancomycin application might be more likely. Furthermore, we excluded high-risk patients with prior AKI episodes or chronic kidney disease not on dialysis, which might have introduced selection bias. This patient group represents the most vulnerable subjects towards drug-induced AKI and it’s likely that such subjects are treated with other substances instead of vancomycin. We argue that this might bias the effect measure estimate towards the null, since the vancomycin group might have a lower baseline risk of observing the outcome, compared to the comparator groups.

Third, linezolid, the most appropriate comparator antibiotic for vancomycin, was scarcely used compared to vancomycin, possibly due to its high cost. Subsequently, we included other comparators, some of which may be less suitable such as beta-lactams. However, all comparators are plausible choices for empirical treatment of early-stage sepsis and are comparable in indication. Separate effect estimates are reported for each comparator.

Fourth, we could not account for antibiotic combination treatments with aminoglycosides, because HCPC codes are not available for those treatments. Furthermore, we didn’t control for other concomitantly administered nephrotoxic agents, such as radio contrasts or chemotherapies, and detailed data on oral medication during hospitalization is not available in claims data. However, it’s plausible to assume that administration of such agents did not differ across treatment groups in this large sample. Furthermore, we excluded more than 15,000 (17%) patients with concomitant administration of the seven antibiotics used in the analysis, which represent the most frequently used types of IV antibiotic combination treatments^1^.

Fifth, residual confounding may remain. More precise definitions on treatment indication (“main diagnosis”) would have further informed the analysis, but diagnosis codes in claims data are known to be less informative compared to clinical chart data^50^. However, we accounted for various surgical procedures with a known increased incidence of postoperative AKI and ICU stay, which might have more effectively controlled for confounding than specific types of infections would do. Furthermore, laboratory measures would have helped to further differentiate the distribution of patients with a more severe grade of acute illness across groups. By the nature of claims data this information is not available.

In conclusion, our cohort study does not provide evidence of increased risk of AKI in vancomycin mono-treated patients compared to patients treated with other commonly used IV antibiotics in hospitalized patients with similar clinical indications in a lager U.S. commercial claims database.

## Supplementary information

Supplementary file1

## Data Availability

The data that support the findings of this study are available from MaketScan Research Databases (IBM Watson Health) but restrictions apply to the availability of these data, which were used under license for the current study, and so are not publicly available.
