# Supplementary material for: The comparative risk of acute kidney injury of vancomycin relative to other common antibiotics
Source: Sci Rep. 2020 Oct 14;10:17282. doi: 10.1038/s41598-020-73687-9 (PMC7560595; doi:10.1038/s41598-020-73687-9)
Supplement: Supplementary file 1 — Supplementary file1 [file 41598_2020_73687_MOESM1_ESM.docx]

**Supplemental Material**

The Comparative Risk of Acute Kidney Injury of Vancomycin Relative to other Common Antibiotics

Gaggl M,^1,2^ Pate V,^1^ Stürmer T,^1^ Kshirsagar AV,^3^ and Layton JB^1,4^

^1^Department of Epidemiology, University of North Carolina at Chapel Hill, Chapel Hill, North Carolina, USA.

^2^Division of Nephrology and Dialysis, Department of Medicine III, Medical University of Vienna, Vienna, Austria.

^3^UNC Kidney Center and Division of Nephrology & Hypertension, University of North Carolina at Chapel Hill, Chapel Hill, North Carolina, USA.

^4^RTI Health Solutions, Research Triangle Park, North Carolina, USA.

Corresponding Author

Martina Gaggl

Department of Epidemiology

Gillings School of Global Public Health

135 Dauer Drive

2101 McGavran-Greenberg Hall, CB #7435

University of North Carolina at Chapel Hill

Chapel Hill, North Carolina, USA

Division of Nephrology and Dialysis

Department of Medicine III

Währinger Gürtel 18-20

Medical University of Vienna

Vienna, Austria, EU

Mail: [martina.gaggl@meduniwien.ac.at](mailto:martina.gaggl@meduniwien.ac.at)

Tel.: +43 1 40400 43910

Supplemental table 1a. Exposure

|  | **Codes (HCPC)** | **Effective date** |
| --- | --- | --- |
| Vancomycin | J3370 | 01/01/1986 |
| **Comparators** |  |  |
| *Similar bactericidal spectrum* |  |  |
| Cefazolin | J0690 | 01/01/1982 |
| Linezolid | J2020 | 01/01/2002 |
| Daptomycin | **J0878, C9124** | 01/01/2004 |
| *Similar clinical indications* |  |  |
| Piperacillin+ Tazobactam | J2543, S0081 | 01/01/2000 |
| Cefepime | J0692 | 01/01/2002 |
| Meropenem | **J2185** | 01/01/2004 |
| Ertapenem | **C9116, J1335** | 10/01/2002 |

Supplemental table 1b. Outcome

|  | **Codes (ICD-9)** | **Effective date** |
| --- | --- | --- |
| Acute kidney injury | 584.5, 584.6, 584.7, 584.8, 584.9, 586, 585.6, 639.3 | 01/01/96 |
| Dialysis | 389.5, 392.7, 394.2, 394.3, 399.5, 549.8 |  |

Supplemental table 1c. Covariates

|  | **Codes (ICD-9)** |
| --- | --- |
| Cardiovascular disease | 410, 410.0, 410.00, 410.01, 410.02, 410.1, 410.10, 410.11, 410.12, 410.2, 410.20, 410.21, 410.22, 410.3, 4103.0, 410.31, 410.32, 410.4, 410.40, 41041, 410.42, 410.5, 410.50, 410.51, 410.52, 410.6, 410.60, 410.61, 410.62, 410.7, 410.70, 410.71, 410.72, 410.8, 410.80, 410.81, 410.82, 410.9, 410.90, 410.91, 410.92, 414, 414.8, 429, 429.7, 648, 648.5, 648.6 |
| Arterial fibrillation | 427.3, 427.31,427.32 |
| Other heart disease | 93, 938, 938.9, 391, 391.8, 392, 392.0, 398, 398.9, 39891, 402, 402.0, 402.00, 402.01, 40.21, 402.10, 402.11, 402.9, 402.90, 402.91, 404, 404.0, 404.00, 404.01, 404.02, 404.03, 404.1, 404.10, 404.11, 404.12, 404.13, 404.9, 404.90, 404.91, 404.92, 404.93, 428, 428.0, 428.1, 428.2, 428.20, 428.21, 428.22, 428.23, 428.3, 428.30, 428.31, 42832, 428.33, 428.4, 428.40, 428.41, 428.42, 428.43, 428.9, 429, 429.1, 429.4, 440, 440.9, 639, 639.8, 668, 668.1, 669, 669.4, 779, 779.8, 779.89, 797, 995, 995.9, 995.92, 995.94, 996, 996.8, 996.83, 997, 997.1, E872, E8725, E8726, E874, E8744, E8745 |
| Chronic kidney disease | 585.1, 585.2, 585.3, 585.4, 585.5, 585.6, 585.9 |
| Renal replacement therapy | 389.5, 392.7, 394.2, 394.3, 399.5, 549.8 |
| Renal transplantation | 556 |
| Hyperlipidemia | 272, 272.0, 272.1, 272.2, 272.3, 272.4, V77, V779, V779.1 |
| Nephrolithiasis | 274, 274.1, 274.11, 592, 592.0 |
| Diabetes | 249, 249.0, 249.00, 249.01, 249.1, 249.10, 249.11, 249.2, 249.20, 492.1, 249.3, 249.30, 249.31, 249.4, 249.40, 249.41, 249.5, 249.50, 249.51, 249.6, 249.60, 249.61, 249.7, 249.70, 249.71, 249.8, 249.80, 249.81, 249.9, 249.90, 249.91, 250, 250.0, 250.00, 250.01, 250.02, 250.03, 250.1, 250.10, 250.11, 250.12, 250.13, 250.2, 250.20, 250.21, 250.22, 250.23, 250.3, 250.30, 250.31, 250.32, 250.33, 250.4, 250.40, 250.41, 250.42, 250.43, 250.5, 250.50, 250.51, 250.52, 250.53, 250.6, 250.60, 250.61, 250.62, 250.63, 250.7, 250.70, 250.71, 250.72, 250.73, 250.8, 250.80, 250.81, 250.82, 250.83, 250.9, 250.90, 250.91, 250.92, 250.93, 251, 251.3, 251.8, 519, 253, 253.5, 258, 258.1, 271, 271.4, 275, 275.0, 275.01, 275.03, 275.3, 337, 337.1, 353, 353.5, 357, 357.2, 358, 358.1, 443, 443.8, 443.81, 536, 536.3, 581, 581.8, 581.81, 583, 583.8, 583.81, 588, 588.1, 648, 648.0, 648.00, 648.01, 648.02, 648.03, 648.04, 648.8, 707, 707.1, 731, 731.8, 775, 775.0, 775.1, 790, 790.2, 790.29, 962, 962.0, V12, V122, V122.1, V18, V180, V65, V653, V77, V771, V88, V881 |
| Lupus | 017, 017.0, 135, 286, 286.5, 286.53, 289, 289.8, 289.81, 357, 357.1, 359, 359.6, 373, 373.3, 373.34, 373.4, 424, 424.9, 424.91, 517, 517.8, 581, 581.8, 581.81, 582, 582.8, 582.81, 583, 583.8, 583.81, 695, 695.4, 710, 710.0, 795, 795.7, 795.79, 972, 972.6, 991, 991.5 |
| Liver disease | 095, 095.3, 275, 275.0, 275.01, 275.1, 303, 437, 437.0, 456, 456.2, 571, 571.0, 571.2, 571.5, 571.8, 573, 573.5, 777, 777.8 |
| Myeloma | 203, 203.0, 203.00, 203.01, 203.02, 238,  238.6, 713, 713.2, V16, V167 |
| Hypertension | 093, 093.8, 093.82, 306, 306.2, 348, 348.2, 365, 365.0, 365.04, 401, 401.9, 402, 402.0, 402.00, 402.1, 402.10, 402.9, 402.90, 402.91, 403, 403.9, 403.90, 403.91, 404, 404.9, 404.90, 404.91, 404.92, 404.93, 405, 405.9, 405.91, 405.99, 410, 410.0, 410.1, 410.2, 410.3, 410.4, 410.5, 410.6, 410.7, 410.8, 410.9, 411, 411.1, 412, 414, 414.8, 415, 415.0, 416, 416.0, 416.8, 424, 424.3, 424.9, 424.99, 437, 437.2, 440, 440.1, 456, 456.2, 459, 459.1, 459.10, 459.3, 459.30, 459.31, 459.32, 459.33, 459.39, 572, 572.3, 577, 577.1, 642, 642.0, 642.00, 642.01, 642.02, 642.03, 642.04, 642.1, 642.10, 642.11, 642.12, 642.3, 642.14, 642.2, 642.20, 642.21, 642.22, 642.23, 642.24, 642.3, 642.30, 642.31, 642.32, 642.33, 642.34, 642.4, 642.5, 642.7, 642.70, 642.71, 642.72, 642.73, 642.74, 642.9, 642.90, 642.91, 642.92, 642.93, 642.94, 646, 646.1, 646.10, 646.11, 646.12, 646.13, 646.14, 646.2, 646.20, 646.21, 646.22, 646.23, 646.24, 707, 707.1, 747, 747.8, 747.83, 760, 760.0, 796, 796.2, 997, 997.1, 997.9, 997.91, V17, V174, V174.9 , V81, V811 |
| Hypercalcemia | 269, 269.3, 275, 275.4, 275.40, 275.41, 275.42, 275.49, 379, 379.2, 379.22, 579, 579.8, 727, 727.8, 727.82, 961, 961.8, 961.9, 963, 963.5, 963.8, 965, 965.1, 973, 973.0, 973.2, 974, 974.1, 974.5, 977, 977.3, 983, 983.2, 983.9, 987, 987.8 |
| Co-medication | Angiotensin-converting enzyme inhibitors, angiotensin II receptor antagonists, statins, beta-blockers, calcium channel blockers, antiplatelet agents, alpha-blockers, thiazide, K+-sparing diuretics, loop diuretics, niacin, fibrates, ezetimibe, anticoagulants, non-steroidal anti-inflammatory drugs (NSAID), steroids, tacrolimus, sirolimus, cyclosporin A, mycophenolate mofetil  Due to the extent of individual NDC codes complete definitions are not presented. Complete definitions are shared upon request. |

Supplemental table 1d. Main diagnosis (CCS-procedure code classification)[^6^](#_ENREF_6)

| **Main Diagnosis** | **CCS-DX** | **Diagnosis** |
| --- | --- | --- |
| Sepsis | 2 | Septicemia |
|  | 246 | Fever |
| Skin infection | 197 | Skin infections |
|  | 247 | Lymphadenitis |
|  | 190 | Ulcers |
| Other infection | 1, 3, 9 | Bacterial infections |
|  | 8, 4 | Mycoses and parasitic disease |
|  | 159 | Urinary tract infection |
|  | 5-7 | Viral infections |
|  | 76-78 | Meningitis and Encephalitis |
|  | 135 | Intestinal infection |
|  | 90,92 | Ear and eye infection |
|  | 122-127 | Respiratory infections |
| Disease of circulatory system | 96-108 | Disease of the heart |
|  | 109-113 | Cerebrovascular disease |
|  | 114-117 | Disease of arteries |
|  | 118-121 | Disease of veins and lymphatic |
| Respiratory disease | 127-134 | Respiratory infections |
| Gastrointestinal disease | 136-155 | Disease of the digestive system |
| Cancer | 11-47 | Neoplasms |
| Disease of the genitourinary system | 156-175 |  |
| Disease of the musculoskeletal system and connective tissue | 201-212 |  |
| Injury & Poisoning | 225-244 | Injury and poisoning |
| Metabolic disease | 48-58 |  |

Supplemental table S1e. Definitions of types of surgeries[^46^](#_ENREF_46)

| **Type of Surgery** | **CCS-PC** | **Surgery** |
| --- | --- | --- |
| ENT | 10 | Thyroidectomy; partial or complete |
|  | 30 | Tonsillectomy and/or adenoidectomy |
|  | 33 | Other OR therapeutic procedures on nose; mouth and pharynx |
| Thoracic | 36 | Lobectomy or pneumonectomy |
|  | 42 | Other OR Rx procedures on respiratory system and mediastinum |
|  | 167 | Mastectomy |
|  | 175 | Other OR therapeutic procedures on skin and breast |
| Cardiac | 43 | Heart valve procedures |
|  | 44 | Coronary artery bypass graft (CABG) |
|  | 49 | Other OR heart procedures |
|  | 50 | Extracorporeal circulation auxiliary to open heart procedures |
| Vascular | 51 | Endarterectomy; vessel of head and neck |
|  | 52 | Aortic resection; replacement or anastomosis |
|  | 53 | Varicose vein stripping; lower limb |
|  | 54 | Other vascular catheterization; not heart |
|  | 55 | Peripheral vascular bypass |
|  | 56 | Other vascular bypass and shunt; not heart |
|  | 59 | Other OR procedures on vessels of head and neck |
|  | 60 | Embolectomy and endarterectomy of lower limbs |
|  | 61 | Other OR procedures on vessels other than head and neck |
| General | 66 | Procedures on spleen |
|  | 71 | Gastrostomy; temporary and permanent |
|  | 72 | Colostomy; temporary and permanent |
|  | 73 | Ileostomy and other enterostomy |
|  | 74 | Gastrectomy; partial and total |
|  | 75 | Small bowel resection |
|  | 80 | Appendectomy |
|  | 84 | Cholecystectomy and common duct exploration |
|  | 85 | Inguinal and femoral hernia repair |
|  | 86 | Other hernia repair |
|  | 89 | Exploratory laparotomy |
|  | 90 | Excision; lysis peritoneal adhesions |
|  | 94 | Other OR upper GI therapeutic procedures |
|  | 96 | Other OR lower GI therapeutic procedures |
|  | 99 | Other OR gastrointestinal therapeutic procedures |
|  | 113 | Transurethral resection of prostate (TURP) |
|  | 114 | Open prostatectomy |
|  | 118 | Other OR therapeutic procedures; male genital |
| Ortho | 142 | Partial excision bone |
|  | 153 | Hip replacement; total and partial |
|  | 154 | Arthroplasty other than hip or knee |
|  | 157 | Amputation of lower extremity |
|  | 158 | Spinal fusion |
|  | 161 | Other OR therapeutic procedures on bone |
|  | 162 | Other OR therapeutic procedures on joints |
|  | 164 | Other OR therapeutic procedures on musculoskeletal system |
| Minor | - | If label of procedure code includes either term: “amputation”, “fusion”, “repair”, “resection”, “laceration”, “_tomy”, “_plasty”; “resection”, “excision”, “revision”, “fixation”, “reconstruction”, “anastomosis”, “operation”, “reduction”, “_pexy”, “open” |

Minor, subjects with any of the indicated terms included in the procedure label, but classified with any of the other surgery type classes.

CCS-PM, CCS procedure codes; ENT, ear-nose-throat.

Supplemental table 2. Main diagnosis and type of surgical procedure with respect to antibiotic treatment.

|  | |  | **Vancomycin** | | **Cefazolin** | | **Linezolid** | | **Piperacillin** | | **Cefepime** | | **Meropenem** | | **Ertapenem** | | **Daptomycin** | |
| --- | --- | --- | --- | --- | --- | --- | --- | --- | --- | --- | --- | --- | --- | --- | --- | --- | --- | --- |
| Gender (female) | | | 2,945 | 14.9% | 12,733 | 64.3% | 124 | 0.6% | 2,124 | 10.7% | 232 | 1.2% | 203 | 1.0% | 1,305 | 6.6% | 134 | 0.68% |
| Age (years) | | | 50.4 | ±15.7 | 50.9 | ±14.6 | 49.7 | ±16.8 | 47.8 | ±16.03 | 48.4 | ±18.7 | 48.6 | ±14.6 | 48.6 | ±13.2 | 50.4 | ±13.4 |
| *Treatment duration* | | |  |  |  |  |  |  |  |  |  |  |  |  |  |  |  |  |
|  | | ≤7 days | 4,347 | 79.8% | 18,219 | 90.5% | 103 | 41.%0 | 3,125 | 80.4% | 295 | 68.1% | 243 | 64.1% | 1,802 | 81.7% | 178 | 65.7% |
|  | | 8-14 days | 871 | 16.0% | 1,594 | 7.9% | 66 | 26.3% | 597 | 15.4% | 91 | 21.0% | 93 | 24.5% | 330 | 15.0% | 74 | 27.3% |
|  | | ≥15 days | 231 | 4.2% | 311 | 1.5% | 82 | 32.7% | 163 | 4.2% | 47 | 10.9% | 43 | 11.3% | 73 | 3.3% | 19 | 7.0% |
| *Main diagnosis* | | |  |  |  |  |  |  |  |  |  |  |  |  |  |  |  |  |
|  | Sepsis | | 372 | 6.8% | 54 | 0.3% | 61 | 24.3% | 286 | 7.4% | 47 | 10.9% | 56 | 14.8% | 80 | 3.6% | 30 | 11.1% |
|  | Skin infection | | 968 | 17.8% | 156 | 0.8% | 38 | 15.1% | 121 | 3.1% | 11 | 2.5% | 8 | 2.1% | 46 | 2.1% | 92 | 33.9% |
|  | Other infection | | 900 | 16.5% | 516 | 2.6% | 67 | 26.7% | 647 | 16.7% | 141 | 32.6% | 93 | 24.5% | 178 | 8.1% | 53 | 19.6% |
|  | Respiratory disease | | 94 | 1.7% | 154 | 0.8% | 10 | 4.0% | 107 | 2.8% | 29 | 6.7% | 13 | 3.4% | 9 | 0.4% | 1 | 0.4% |
|  | Gastrointestinal disease | | 147 | 2.7% | 1,148 | 5.7% | 12 | 4.8% | 1,813 | 46.7% | 30 | 6.9% | 108 | 28.5% | 985 | 44.7% | 4 | 1.5% |
|  | Genitourinary disease | | 64 | 1.2% | 1,668 | 8.3% | 4 | 1.6% | 84 | 2.2% | 13 | 3.0% | 7 | 1.8% | 51 | 2.3% | 4 | 1.5% |
|  | Cancer | | 197 | 3.6% | 3,445 | 17.1% | 12 | 4.8% | 156 | 4.0% | 57 | 13.2% | 27 | 7.1% | 407 | 18.5% | 6 | 2.2% |
|  | Circulatory disease | | 528 | 9.7 | 1,358 | 6.7% | 17 | 6.8% | 133 | 3.4% | 20 | 4.6% | 14 | 3.7% | 20 | 0.9% | 9 | 3.3% |
|  | Metabolic disease | | 208 | 3.8% | 1,187 | 5.9% | 5 | 2.0% | 128 | 3.3% | 15 | 3.5% | 11 | 2.9% | 250 | 11.3% | 10 | 3.7% |
|  | Musculoskeletal disease | | 1,202 | 22.1% | 6,465 | 32.1% | 4 | 1.6% | 12 | 0.3% | 17 | 3.9% | 2 | 0.5% | 11 | 0.5% | 13 | 4.8% |
|  | Injury & poisoning | | 490 | 9.0% | 2,637 | 13.1% | 15 | 6.0% | 210 | 5.4% | 21 | 4.8% | 23 | 6.1% | 117 | 5.3% | 39 | 14.4% |
|  | Other | | 279 | 5.1% | 1,336 | 6.6% | 6 | 2.4% | 188 | 4.8% | 32 | 7.4% | 17 | 4.5% | 51 | 2.3% | 10 | 3.7% |
| *Surgical procedure* | | |  |  |  |  |  |  |  |  |  |  |  |  |  |  |  |  |
|  | | Cardiac | 316 | 5.8% | 616 | 3.1% | 1 | 0.4% | 9 | 0.2% | 3 | 0.7% | 6 | 1.6% | 1 | 0.05% | 3 | 1.1% |
|  | | Thoracic | 83 | 1.5% | 567 | 2.8% | 13 | 5.2% | 17 | 0.4% | 4 | 0.9% | 0 |  | 9 | 0.4% | 0 |  |
|  | | Vascular | 87 | 1.6% | 410 | 2.0% | 7 | 2.8% | 21 | 0.5% | 3 | 0.7% | 6 | 1.6% | 9 | 0.4% | 4 | 1.5% |
|  | | ENT | 29 | 0.5% | 107 | 0.5% | 1 | 0.4% | 10 | 0.3% | 1 | 0.2% | 1 | 0.3% | 2 | 0.1% | 1 | 0.4% |
|  | | General | 139 | 2.6% | 2,116 | 10.5% | 30 | 12.0% | 1,215 | 31.3% | 18 | 4.2% | 54 | 14.2% | 1,054 | 47.8% | 4 | 1.5% |
|  | | Ortho | 901 | 16.5% | 3,770 | 18.7% | 5 | 2.0% | 20 | 0.5% | 10 | 2.3% | 2 | 0.5% | 16 | 0.7% | 31 | 11.4% |
|  | | Urology | 2 | 0.04% | 249 | 1.2% | 0 |  | 4 | 0.1% | 1 | 0.2% | 3 | 0.8% | 1 | 0.05% | 0 |  |
|  | | Minor | 408 | 7.5% | 3,028 | 15.0% | 18 | 7.2% | 93 | 2.4% | 17 | 3.9% | 17 | 4.5% | 153 | 6.9% | 34 | 12.5% |
| No surgical procedure | | | 3,484 | 63.9% | 9,261 | 46.0% | 176 | 70.1% | 2,496 | 64.2% | 376 | 86.8% | 290 | 76.5% | 960 | 43.5% | 194 | 71.6% |
|  | | **All** | 5,449 | 16.5% | 2,0124 | 61.0% | 251 | 0.8% | 3,885 | 11.8% | 433 | 1.3% | 379 | 1.1% | 2,205 | 6.7% | 271 | 0.8% |
